# Clinical and Visual Outcomes of Four Presbyopia Correcting Intraocular Lenses

**DOI:** 10.18502/jovr.v19i2.11034

**Published:** 2024-06-21

**Authors:** Pruthvi K. Sharlin, Saumya Patel, Victoria C. Kuritza, Nichole Pompey, Kayéromi D. Gomez, Mitul R. Vakharia

**Affiliations:** ^1^Duly Health and Care, Naperville, IL; ^2^Department of Ophthalmology, University of Illinois College of Medicine, Rockford, IL; ^3^Department of Dermatology, University of Illinois Hospital, Chicago, IL; ^5^: https://orcid.org/0000-0002-5609-0426

**Keywords:** Cataracts, Halos, IOLs (Intraocular Lens), Photic Phenomena, Visual Acuity

## Abstract

**Purpose:**

To compare objective and subjective outcomes of the multifocal intraocular lenses ReSTOR SN6AD1 and Tecnis ZKB00, extended depth of focus IOL Symfony ZXR00, and trifocal IOL PanOptix TFNT00.

**Methods:**

This study included 262 patients (524 eyes) who had phacoemulsification with IOL implantation, 128 eyes with SN6AD1, 124 eyes with ZKB00, 136 eyes with ZXR00, and 136 eyes with TFNT00. Objective outcomes included one-month postoperative uncorrected (U) and corrected (C) distance (D) and near (N) visual acuities (VA). Subjective outcomes included photic phenomena, spectacle use, and spectacle-independent visual function.

**Results:**

Spectacle use (%) in the SN6AD1, ZKB00 ZXR00, and TFNT00 groups was 39, 64, 87, and 37 respectively (*P* < 0.0001). Presence of photic phenomena (%) for SN6AD1, ZKB00, ZXR00, and TFNT00 was 66, 61, and 67, and 73, respectively (*P* = 0.57). Spectacle-independent mean VF-14 score (%) for SN6AD1, ZKB00, ZXR00, and TFNT00 was 89.5, 87.2, 80.9, and 83.6, respectively (*P* < 0.01).

**Conclusion:**

All four IOLs provided excellent postoperative visual acuity and equally high rates of photic phenomena. SN6AD1 and TFNT00 provided the least spectacle use while ZXR00 had the highest spectacle use.

##  INTRODUCTION

Cataract is the leading cause of blindness globally, and unsurprisingly, cataract surgery is the most common surgical procedure performed worldwide. Modern surgical techniques to remove a cataract and replace it with an intraocular lens (IOL) implant allow for nearly complete restoration of normal visual function.^[1]^ Traditionally, patients have received monofocal IOLs which provide adequate distance visual acuity. However, studies show that patients remain unsatisfied due to spectacle dependence for near vision after cataract surgery.^[2]^

Technological advancements have guided the development of new multifocal (MF) IOLs, and more recently, extended depth of focus (EDOF) IOLs and trifocal IOLs. These provide a greater range of vision by allowing improved near vision without compromising distance vision. Patients show higher overall levels of satisfaction in objective and subjective measures, with spectacle independence playing a major role in the improvement of vision-related quality of life.^[2,3]^

Yet better near visual acuity with IOLs may be accompanied by photic phenomena such as halos and glare.^[4]^ Halos are bright circles that occur around sources of light caused by the overlapping of an unfocused near image and focused distance image on the retina. Halos can appear around car headlights and streetlights often affecting nighttime driving. Halo size increases with increasing pupil size, therefore explaining its incidence in low-light conditions.^[5]^ Glare is caused by unfocused light that enters the eye and can also impair night vision. MF lens have higher rates of these photic phenomena than monofocal IOLs.^[6]^ Over time, most patients adapt and the impact of the halos and glare diminish.^[7]^

ReSTOR SN6AD1 is an MF IOL with a central zone of concentric diffractive rings which provide an add power of 3 D at the spectacle plane for near vision.^[8]^ Tecnis ZKB00 is an MF IOL with diffractive rings to the periphery of the optic, theoretically making it more pupil-independent, providing an add power of 2.01 D at the spectacle plane.^[9]^ Symfony ZXR00 is an EDOF IOL which uses diffractive echelettes to create a pupil-independent elongated focus and provides continuous vision from distance through a near add of 1.75 D.^[10]^ PanOptix TFNT00 is a trifocal IOL which uses a non-apodised diffractive zone to provide continuous vision from distance through a near add of 3.25 D.^[11]^ All of these IOLs are single piece foldable aspheric hydrophobic acrylic to compensate for corneal spherical aberration. A lower add power improves intermediate vision and reduces photic phenomena.^[12,13]^

Objective measures alone such as VA are not sufficient to assess visual function after IOL implantation. Therefore, the VF-14 questionnaire was designed to subjectively evaluate functional capacity of patients based on 14 vision-dependent activities that may be affected by cataracts and are expected to improve after replacement with an IOL. Studies suggest that the VF-14 is a reliable indicator of self-reported visual function.^[14]^ This questionnaire was used to assess spectacle-independent visual function for each of the IOLs, and included additional questions regarding postoperative spectacle use and photic phenomena.

##  METHODS

A retrospective chart review (code of ethics: 20160048) and subsequent prospective comparative study was performed. Institutional Review Board approval was obtained from the University of Illinois College of Medicine in Rockford. A total of 262 patients (524 eyes) aged 28–85 years (mean age, 66.80 ± 8.33 years) at the New Vision Laser Center in Rockford, Illinois were included: 64 patients (128 eyes) with SN6AD1, 62 patients (124 eyes) with ZKB00, 68 patients (136 eyes) with ZXR00, and 68 patients (136 eyes) with TFNT00. All patients were part of a consecutive case cohort, thereby eliminating selection bias. The first cohort were only offered SN6AD1 from 4/2009 to 12/2014, the second cohort were only offered ZKB00 from 3/2015 to 8/2016, the third cohort were only offered ZXR00 from 4/2017 to 3/2018, and the fourth cohort were only offered TFNT00 from 9/2019 to 6/2020. Preoperative cataract grading, preoperative CDVA, one month postoperative uncorrected and corrected DVA and NVA, and latest postoperative UDVA were collected from the electronic and paper medical records.

**Figure 1:**
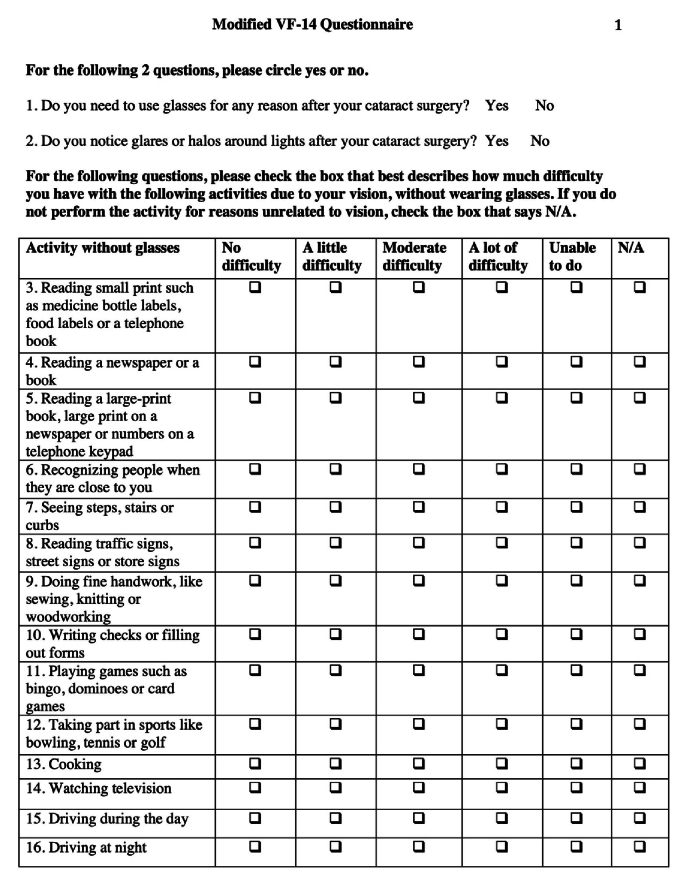
Modified Visual Function Index (VF-14) questionnaire.

Spectacle dependence, photic phenomena, and spectacle-independent visual function were assessed with a modified VF-14 survey, as shown in Figure 1. There was an overall 84% questionnaire response rate, with 56 out of 64 responses for SN6AD1, 59 out of 62 responses for ZKB00, 53 out of 68 responses for ZXR00, and 54 out of 68 responses for TFNT00. Moreover, 222 out of 262 questionnaires were filled out, and 40 were not returned.Average follow-up time from implantation of the IOL to questionnaire response for SN6AD1, ZKB00, ZXR00, and TFNT00 was 1591, 324, and 266 days, and 346 days, respectively.

### Inclusion/Exclusion Criteria

Patients above the age of 18 were eligible for surgery if they had cataract in one or both eyes or had refractive error and opted for a clear lens exchange, and lack of other ophthalmological diseases such as post-transplant cornea, diabetic retinopathy, irregular astigmatism, macular degeneration, chronic uveoscleritis, damage to ciliary/zonular system of lens, retinal detachment, glaucoma, or optic neuropathy. Patients were only offered SN6AD1 or ZKB00 if they had < 1 D of corneal astigmatism, whereas ZXR00 and TFNT00 which came in toric models were used for corneal astigmatism > 1 D.

### Multifocal Intraocular Lens Characteristics

The Acrysof IQ ReSTOR IOL (SN6AD1; Alcon Laboratories, Inc. [6201 South Freeway Fort Worth, TX 76134-2099 USA]) is a single-piece MF lens with +3.00 D add power in the IOL plane. The anterior surface contains a central 3.6 mm apodised diffractive zone with nine concentric rings of gradually decreasing height designed to create near and distance foci. It also contains an outer refractive zone designed to direct light to a distance focal point. The IOL has a symmetric biconvex design with a –0.1 μ m anterior aspheric surface designed to reduce spherical aberration of the cornea. It is made of UV and blue light filtering acrylate/methacrylate copolymer material.

The Tecnis Multifocal IOL (ZKB00; Abbott Medical Optics, Inc. [1700 East St. Andrew Place Santa Ana, CA 92705- 4933]) is a single-piece MF lens with +2.75 D add power. It has an anterior aspheric surface (–0.27 μ m) designed to compensate for corneal spherical aberration. The IOL also has a 6-mm full-aperture diffractive posterior surface with 15 concentric rings created to provide near and distance vision. ZKB00 is made of UV-blocking hydrophobic acrylic material. The posterior edge of the optic has a 360º-squared design to prevent posterior capsule opacification.

**Table 1:** Preoperative characteristics in patients undergoing cataract extraction and bilateral implantation of intraocular lenses SN6AD1, ZKB00, ZXR00, and TFNT00


**Characteristics**	**Overall**	**SN6AD1 (1)**	**ZKB00 (2)**	**ZXR00 (3)**	**TFNT00 (4)**	***P-*** **value** ${\;}^{†}$	***P-*** **value post hoc comparison** ${\;}^{‡}$					
			* *	**1 to 2**	**1 to 3**	**1 to 4**	**2 to 3**	**2 to 4**	**3 to 4**	
Sample size (number of eyes)	262 (524)	64 (128)	62 (124)	68 (136)	68 (136)				
Female, %	51	45.3	51.6	55.9	64.7				
Age, yr Mean ± SD	66.80 ± 8.33	65.13 ± 8.25	65.87 ± 10.6	67.6 ± 7.04	68.26 ± 6.93	0.2	0.79	0.31	0.17	0.93	0.79	1.00	
Range, median	47–80, 66	28–84, 67	47–85, 68	28–85, 67				
Cataract grade, eyes ± SD	2.05 ± 0.030	1.79 ± 0.93	1.98 ± 0.70	2.24 ± 0.53	2.18 ± 0.42	< 0.001 ${\;}^{*}$	0.42	< 0.001 ${\;}^{*}$	< 0.01 ${\;}^{*}$	0.03 ${\;}^{*}$	0.15	0.71	
0	22	16	6	0	0				
1	42	23	13	6	2				
2	236	61	82	93	105				
3	87	28	23	36	29				
4	1	0	0	1	0	5	4	1	6	3	2	
Preoperative CDVA ± SD	0.15 ± 0.15	0.18 ± 0.15	0.24 ± 0.22	0.22 ± 0.17	< 0.001 ${\;}^{*}$	0.22	< 0.001 ${\;}^{*}$	< 0.01 ${\;}^{*}$	0.19	0.37	0.97	
	
	
${\;}^{*}$ Statistical significance (*P* < 0.05); ${\;}^{†}$ Calculated with the Kruskal–Wallis test; ${\;}^{‡}$ Dwass–Steel–Critchlow–Fligner pairwise comparison CDVA, corrected distance visual acuity; SD, standard deviation													

**Table 2:** Corrected and uncorrected distance and near visual acuities one month post-operation and latest uncorrected distance visual acuity, in logMAR, after cataract extraction and bilateral implantation of intraocular lenses SN6AD1, ZKB00, ZXR00, or TFNT00


**Timeframe**	**Parameter**	**SN6AD1 (1) (logMAR ± SD)**	**ZKB00 (2) (logMAR ± SD)**	**ZXR00 (3) (logMAR ± SD)**	**TFNT00 (4) (logMAR ± SD)**	***P-*** **value** ${\;}^{†}$ * *	***P-*** **value post hoc comparison** ${\;}^{‡}$					
			* *	**1 to 2**	**1 to 3**	**1 to 4**	**2 to 3**	**2 to 4**	**3 to 4**	
One month postoperative VA	CDVA	0.027 ± 0.068	0.031 ± 0.076	0.051 ± 0.10	0.094 ± 0.084	< 0.001 ${\;}^{*}$	1.00	0.07	< 0.001 ${\;}^{*}$	0.11	< 0.001 ${\;}^{*}$	< 0.01 ${\;}^{*}$	
	CNVA	0.0060 ± 0.028	0.0094 ± 0.029	0.065 ± 0.13	0.088 ± 0.099	< 0.001 ${\;}^{*}$	0.76	< 0.01 ${\;}^{*}$	< 0.001 ${\;}^{*}$	0.02 ${\;}^{*}$	< 0.001 ${\;}^{*}$	0.39	
	UDVA	0.11 ± 0.12	0.099 ± 0.14	0.099 ± 0.14	0.15 ± 0.12	< 0.01 ${\;}^{*}$	0.37	0.47	0.41	1.00	< 0.01 ${\;}^{*}$	< 0.01 ${\;}^{*}$	
	UNVA	0.022 ± 0.054	0.043 ± 0.074	0.11 ± 0.15	0.082 ± 0.092	< 0.001 ${\;}^{*}$	0.26	< 0.001 ${\;}^{*}$	< 0.001 ${\;}^{*}$	0.05	0.09	0.89	
Latest post-operative VA	CDVA	0.13 ± 0.12	0.11 ± 0.13	0.097 ± 0.13	0.14 ± 0.10	0.02 ${\;}^{*}$	0.52	0.13	0.96	0.93	0.15	0.03 ${\;}^{*}$	
(One month postoperative) –(Preoperative)	CDVA	–0.11 ± 0.14	–0.15 ± 0.15	–0.18 ± 0.24	–0.12 ± 0.17	0.02 ${\;}^{*}$	0.22	0.02 ${\;}^{*}$	0.97	0.71	0.58	0.14	
	
	
${\;}^{*}$ Statistical significance (*P* < 0.05); ${\;}^{†}$ Calculated with the Kruskal–Wallis test; ${\;}^{‡}$ Dwass–Steel–Critchlow–Fligner pairwise comparison CDVA, corrected distance visual acuity; CNVA, corrected near visual acuity; SD, standard deviation; UDVA, uncorrected distance visual acuity; UNVA, uncorrected near visual acuity													

**Table 3:** Responses to modified VF-14 questionnaire, completed > 3 months after cataract surgery, for subjects with bilateral intraocular lens implants of SN6AD1, ZKB00, ZXR00, or TFNT00


	**SN6AD1 (1)**	**ZKB00 (2)**	**ZXR00 (3)**	**TFNT00 (4)**	***P-*** **value** * *	***P-*** **value post hoc comparison** ${\;}^{§}$ * *					
			* *	**1 to 2**	**1 to 3**	**1 to 4**	**2 to 3**	**2 to 4**	**3 to 4**
Overall VF-14 score (%) ± SD	89.5 ± 13.9	87.2 ± 13.4	80.9 ± 16.3	83.6 ± 16.1	< 0.01 ${\;}^{*†}$	0.39	< 0.01 ${\;}^{*}$	0.03 ${\;}^{*}$	0.09	0.56	0.72
Presence of halos/glares (%)	66.1 (37/56)	61.0 (36/59)	67.3 (35/52)	73.6 (39/53)	0.57 ${\;}^{‡}$			
Spectacle use (%)	39.3 (22/56)	64.4 (38/59)	86.5 (45/52)	37.3 (19/51)	< 0.0001 ${\;}^{*‡}$			
Blank survey responses for halos and spectacle use	8	3	16	17 glasses 15 halo				
	
	
${\;}^{*}$ Statistical significance (*P* < 0.05); ${\;}^{†}$ Calculated with the Kruskal–Wallis test; ${\;}^{‡}$ Calculated with the Cochran-Mantel-Haenszel test; ${\;}^{§}$ Dwass–Steel–Critchlow–Fligner pairwise comparison SD, standard deviation; VF-14, visual function index											

**Figure 2:**
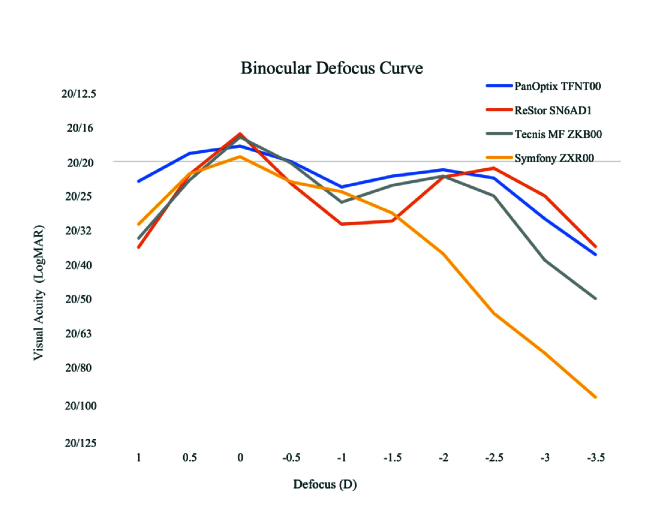
Binocular Defocus Curve: Defocus curves showing visual acuity, in logMAR, at each diopter of defocus for SN6AD1, ZKB00, ZXR00, and TFNT0O.^[15]^

The Symfony IOL (ZXR00; Abbott Medical Optics, Inc. [1700 East St. Andrew Place Santa Ana, CA 92705- 4933]) is a single-piece EDOF lens. It has a biconvex, wavefront-designed –0.27 μ m anterior aspheric surface and 5.5 mm posterior achromatic diffractive surface with nine echelettes. The achromatic technology is designed to correct spherical and chromatic aberration for contrast sensitivity enhancement. The diffractive echelette feature is a proprietary design that elongates a single focal point. The IOL is made of UV-blocking hydrophobic acrylic material. The posterior edge of the optic has a 360º-squared design to prevent posterior capsule opacification.

The PanOptix IOL (TFNT00, Alcon Laboratories, Inc. [6201 South Freeway Fort Worth, TX 76134-2099 USA]) is a single-piece aspheric trifocal lens. It has a 4.5 mm non-apodised diffractive optical structure that allows for three focal points; 50% of the transmitted light is used for distance vision, and 25% each for intermediate vision (+1.00 D add power) and near vision (+3.25 D add power). It uses a hydrophobic acrylate/methacrylate copolymer with a natural chromophore to filter ultraviolet and high-energy visible light.

### Surgical Technique

All procedures were completed by a single surgeon under anesthesia with IV midazolam and fentanyl and topical and intracameral 1% lidocaine (preservative free). A 2.4 mm temporal clear corneal incision was made. Next, phacoemulsification and aspiration of the lens mass were performed via the Infiniti Vision System or Centurion Vision System (Alcon Laboratories, Inc.). An IOL was implanted into the capsular bag with a lens injector, and the incision was closed without sutures. After the procedure, patients were treated with a topical anti-inflammatory medication (prednisolone or difluprednate) and a topical antibiotic (ofloxacin or tobramycin) for four to six weeks. Diabetic patients were also given a topical NSAID. Patients were examined at one day, one-week, and four to six weeks post operation. For each patient, there was a two-week gap between the procedures on each eye. The same IOL model was implanted bilaterally in each patient.

### Statistical Analysis

The results were presented as mean ± standard deviation (SD). All visual acuity measurements were converted to the logarithm of the minimal angle of resolution (logMAR) for data analyses. The Shapiro–Wilk test was performed to evaluate whether the dataset was normally distributed. The Kruskal–Wallis or Cochran–Mantel–Haenszel test compared the four independent study groups. A Dwass–Steel–Critchlow–Fligner pairwise comparison was then performed for the categories that showed a significant difference between the four IOL groups with the Kruskal–Wallis test. Statistical analyses were performed using SAS 9.4 and Jamovi. Statistical significance was defined as *P* < 0.05.

##  RESULTS

Demographic data of patients enrolled in each IOL group are presented in Table 1 along with preoperative clinical data. Two-hundred sixty-two patients (524 eyes) were included in the study. SN6AD1, ZKB00, ZXR00, and TFNT00 had 64, 62, 68, and 68 patients, respectively. There were statistically significant differences among the IOLs in cataract grades (*P* < 0.001), and preoperative CDVA (*P* < 0.001), but not age (*P* = 0.199). The median age was almost identical for all four IOLs. A cataract grade of 3 was most common among all four IOLs. ZXR00 had the highest cataract grade and SN6AD1 had the lowest, and correspondingly, preoperative CDVA was worst for ZXR00 and best for SN6AD1. Pairwise comparisons of the IOLs are also listed in Table 1.

All postoperative VA results are presented in Table 2. There were statistically significant differences among all four IOLs in one-month postoperative CDVA (*P* < 0.001), CNVA (*P* < 0.001), UDVA (*P* < 0.01), UNVA (*P* < 0.001), and latest postoperative UDVA (*P* = 0.02). The difference between preoperative and one-month CDVA were statistically significant (*P* = 0.02). The largest improvement in DVA was in ZXR00 and the lowest improvement was in SN6AD1, corresponding to the worst preoperative CDVA for ZXR00 and best preoperative CDVA for SN6AD1.

Patient-reported postoperative spectacle-independent visual function, presence of halos or glare, and spectacle use are presented in Table 3. Overall, VF-14 scores ranged from 25 to 100%, with a mean of 85.38%. These VF-14 scores indicating spectacle-independent visual function were the highest in SN6AD1, followed by ZKB00, TFNT00, and ZXR00, respectively, and the difference between SN6AD1 and ZXR00 was statistically significant (*P* < 0.01). Among the four IOLs, presence of halos/glare were not statistically significantly different (*P* = 0.57), however, spectacle use was statistically significantly different (*P* < 0.0001), with SN6AD1 and TFNT00 having the least spectacle use.

##  DISCUSSION

Our study revealed that overall self-reported visual function after bilateral implantation of ZXR00, ZKB00, SN6AD1, and TFNT00 was high, and all measurements of postoperative VA for all IOLs were around logMAR 0.1 (20/25) or better. Comparison of changes in the VF-14 score with VA for all IOLs showed that the VF-14 score decreased with decreasing VA, suggesting that spectacle-independent visual function corresponded with visual acuity.

Breakdown of each individual item on the VF-14 questionnaire showed that SN6AD1 patients had the least difficulty with spectacle-independent near vision tasks such as reading small print, doing fine handwork, writing checks, and playing games like cards. This is consistent with the higher add power of the SN6AD1. Our visual acuity data supports this with SN6AD1 showing the best UNVA at one month postoperative. Additionally, SN6AD1 patients also reported low spectacle use.

Although TFNT00 had lower postoperative VA scores which were statistically significant, as all VA measurements for all IOLs were around logMAR 0.1 (20/25) or better, this did not indicate a significant clinical difference. A significantly lower percentage of patients reported spectacle use after surgery with TFNT00 (37%) and SN6AD1 (39%) compared to ZKB00 (64%) and ZXR00 (87%), which led to high spectacle-independent visual function scores for TFNT00 despite differences in VA measurements. This again is likely reflective of the higher add power of TFNT00 and SN6AD1. The defocus curve for TNFT00 shows better intermediate vision than SN6AD1, which was not assessed in this study but is clinically relevant for patients with high computer use [Figure 2].

A significantly higher percentage of patients with ZXR00 reported using glasses after cataract surgery for near vision tasks per survey responses, consistent with this IOL having the lowest add power. The ZKB00 also has a lower add power and therefore also showed higher rates of spectacle use. The ZKB00 had the lowest rate of photic phenomena although this was not statistically significant (*P* = 0.57). The ZXR00 had the worst UNVA of all four IOLs and therefore showed the least spectacle-independent visual function. This is consistent with the defocus curve for ZXR00 which shows worse near visual acuity, dropping to 20/50 at 33 cm [Figure 2].

In this study, we also subjectively evaluated the presence of photic phenomena in patients after bilateral implantation of ZXR00, ZKB00, SN6AD1, or TFNT00. Greater than 60% of patients in all four groups reported that they noticed halos or glare around lights after their cataract surgery and differences in halos and glare among the IOLs were not statistically significant. It should be noted that our study involved directly questioning patients whether they noticed halos and glare which may have led to increased positive responses rather than spontaneous complaints about such phenomena.

This study has some limitations. There was no comparison to a standard monofocal IOL as a control. The study was non-randomized and there was a lack of blinding to IOL type. However, selection bias was limited as patients were part of a consecutive case cohort in which only one type of IOL was being offered at a time for bilateral implantation. Lastly, there was a much larger time span between IOL implantation and VF-14 questionnaire responses for SN6AD1 patients compared to ZKB00, ZXR00, or TFNT00 patients, because the SN6AD1 was available first and surveys were sent to these patients often years after their surgery. It is possible that SN6AD1 patients had higher visual function scores because of more time to adapt to their IOLs. Follow-up in a few years for ZKB00, ZXR00, or TFNT00 patients may reveal improved clinical outcomes after they are able to adapt to these IOLs.

In summary, all four IOLs provided excellent VA postoperatively. SN6AD1 and TFNT00 patients reported the lowest spectacle use. ZXR00 showed the highest spectacle use and the least spectacle-independent visual function. All four groups experienced high rates of photic phenomena, but there was not a statistically significant difference among the four IOLs. Presbyopia-correcting IOL choice should probably not be guided by photic phenomena but rather by postoperative spectacle use which showed a statistically significant difference.

##  Financial Support and Sponsorship

None.

##  Conflicts of Interest

None.

## References

[B1] Asbell PA, Dualan I, Mindel J, Brocks D, Ahmad M, Epstein S (2005). Age-related cataract. Lancet.

[B2] Shah S, Peris-Martinez C, Reinhard T, Vinciguerra P (2015). Visual outcomes after cataract surgery: Multifocal versus monofocal intraocular lenses. J Refract Surg.

[B3] Javitt JC, Steinert RF (2000). Cataract extraction with multifocal intraocular lens implantation: A multinational clinical trial evaluating clinical, functional, and quality-of-life outcomes. Ophthalmology.

[B4] Dick HB, Krummenauer F, Schwenn O, Krist R, Pfeiffer N (1999). Objective and subjective evaluation of photic phenomena after monofocal and multifocal intraocular lens implantation. Ophthalmology.

[B5] Alba-Bueno F, Vega F, Millán MS (2014). [Halos and multifocal intraocular lenses: Origin and interpretation]. Arch Soc Esp Oftalmol.

[B6] Häring G, Dick HB, Krummenauer F, Weissmantel U, Kröncke W (2001). Subjective photic phenomena with refractive multifocal and monofocal intraocular lenses. Results of a multicenter questionnaire J Cataract Refract Surg.

[B7] Rosa AM, Miranda ÂC, Patrício MM, McAlinden C, Silva FL, Castelo-Branco M, et al (2017). Functional magnetic resonance imaging to assess neuroadaptation to multifocal intraocular lenses. J Cataract Refract Surg.

[B8] de Vries NE, Nuijts RM (2013). Multifocal intraocular lenses in cataract surgery: Literature review of benefits and side effects. J Cataract Refract Surg.

[B9] Eom Y, Song JS, Kim HM (2017). Spectacle plane add power of multifocal intraocular lenses according to effective lens position. Can J Ophthalmol.

[B10] Gatinel D, Loicq J (2016). Clinically relevant optical properties of bifocal, trifocal, and extended depth of focus intraocular lenses. J Refract Surg.

[B11] García-Pérez JL, Gros-Otero J, Sánchez-Ramos C, Blázquez V, Contreras I (2017). Short term visual outcomes of a new trifocal intraocular lens. BMC Ophthalmol.

[B12] Kretz FT, Gerl M, Gerl R, Müller M, Auffarth GU; ZKB00 Study Group (2015). Clinical evaluation of a new pupil independent diffractive multifocal intraocular lens with a +2. 75 D near addition: a European multicentre study Br J Ophthalmol.

[B13] Gierek-Ciaciura S, Cwalina L, Bednarski L, Mrukwa-Kominek E (2010). A comparative clinical study of the visual results between three types of multifocal lenses. Graefes Arch Clin Exp Ophthalmol.

[B14] Steinberg EP, Tielsch JM, Schein OD, Javitt JC, Sharkey P, Cassard SD, et al (1994). The VF-14. An index of functional impairment in patients with cataract Arch Ophthalmol.

[B15] Schallhorn JM, Pantanelli SM, Lin CC, Al-Mohtaseb ZN, Steigleman WA 3rd, Santhiago MR, et al (2021). Multifocal and accommodating intraocular lenses for the treatment of presbyopia: A report by the American Academy of Ophthalmology. Ophthalmology.

